# A randomised, double-blind, placebo-controlled crossover trial on cannabinoid-based medication for behavioural disorders in old patients with severe dementia: the study results

**DOI:** 10.1093/ageing/afag239

**Published:** 2026-08-18

**Authors:** Federica Bianchi, Barbara Broers, Jules Alexandre Desmeules, Angeline Langlois, James Wampfler, François Curtin, Sophie Pautex

**Affiliations:** Palliative Medicine Division, Department of Rehabilitation and Geriatrics, Geneva University Hospitals, Geneva, GE, Switzerland; Fondation pour l’accueil et l’hébergement de personnes âgées, Geneva, GE, Switzerland; Faculty of Medicine, University of Geneva, Geneva, GE, Switzerland; Faculty of Medicine, Department of Anesthesiology, Pharmacology, Intensive Care and Emergency Medicine, University of Geneva, Geneva, GE, Switzerland; Fondation pour l’accueil et l’hébergement de personnes âgées, Geneva, GE, Switzerland; Fondation pour l’accueil et l’hébergement de personnes âgées, Geneva, GE, Switzerland; Faculty of Medicine, Department of Anesthesiology, Pharmacology, Intensive Care and Emergency Medicine, University of Geneva, Geneva, GE, Switzerland; Palliative Medicine Division, Department of Rehabilitation and Geriatrics, Geneva University Hospitals, Geneva, GE, Switzerland; Faculty of Medicine, Department of Rehabilitation and Geriatrics, University of Geneva, Geneva, GE, Switzerland

**Keywords:** severe dementia, behavioural and psychological symptoms of dementia, randomised double-blind crossover trial, cannabinoid-based medication, long-term care facilities, older people

## Abstract

**Background:**

Behavioural and psychological symptoms of dementia (BPSD) are frequent in patients with severe dementia and represent a major clinical challenge. Cannabinoid-based medications (CBM) have been proposed as a possible therapeutic option, but clinical evidence is still limited.

**Methods:**

We performed a multicenter, randomised, double-blind, placebo-controlled crossover trial in five long-term care facilities in Geneva, Switzerland. Patients with severe dementia received oral Δ9-tetrahydrocannabinol (THC) and cannabidiol (CBD) oil (1:2 ratio, up to 20 mg/40 mg daily) and placebo in two 8-week periods separated by a 1-week washout. The primary outcome was agitation measured by the Cohen–Mansfield Agitation Inventory (CMAI). Secondary outcomes included behavioural scales, pain, as-needed psychotropic medication use and safety.

**Results:**

Twenty-five patients (average age 83 years) were randomised, and nineteen completed both study periods. No significant difference was observed between active treatment and placebo for the CMAI and other main behavioural outcomes. However, the use of *pro re nata* psychotropic medications was significantly lower during the active treatment period. The treatment was well tolerated, and no serious adverse events related to the study drug were observed.

**Conclusions:**

In this real-life crossover study, THC/CBD oil did not show superiority over placebo in reducing agitation in severe dementia. However, it was safe and associated with a significant reduction in the use of as-needed psychotropic medications prescribed for behavioural and psychological symptoms of dementia. CBM may represent a possible adjunctive option in selected patients, but further studies are needed.

## Key Points

- In this RCT, behavioural symptom improvement with THC/CBD oil did not reach statistical difference versus placebo
- The treatment was well-tolerated with no serious adverse events.
- Use of PRN psychotropic drugs decreased during active treatment.
- Cannabinoids may help selected patients when standard therapies fail.

## Introduction

The increase in life expectancy and an aging population in most countries results in an increase in morbidity, including cognitive disorders [1, 2]. In the context of major neurocognitive disorders and end-of-life care, quality of life and symptom management are core components of patient care and central to high-quality palliative care. This requires careful balancing of therapeutic interventions while considering not only disease-related factors but also the well-being of families, caregivers and healthcare professionals [3]. More than a third of patients with dementia related disorders did not receive palliative care resources despite a high prevalence of symptoms and signs likely to benefit from palliative care [4]. Behavioural and psychological symptoms of dementia (BPSD) constitute a major clinical challenge [5]. Antipsychotics used to manage behavioural and psychological symptoms include of typical and (off label use of) atypical agents; typical antipsychotics have poor tolerability and significant side effects, such as falls, stroke and mortality risk, while atypical antipsychotics act more selectively on dopamine and serotonin receptors and are better tolerated [4], but they don’t have an indication for BPSD except brexpiprazole registered by the Food and Drug Administration (FDA) and by Swissmedic for agitation in Alzheimer’s dementia. Also, patients with dementia are often affected by comorbidities, adding to the complexity of finding the optimal therapy that addresses possible unwanted drug–drug interactions or altered metabolism. Research on aged persons is challenging, and this population is underrepresented in clinical research.

Cannabinoid-based medications (CBM) have been proposed as a possible treatment alternative to relieve the burden of BPSD; however, evidence and research are limited [6]. The effects of cannabinoid-based medications are mainly due to the two major plant derivatives, delta-9-tetrahydrocannabinol (THC) and cannabidiol (CBD), and their active metabolites (11-hydroxy-THC, 7-hydroxy-CBD, 7-carboxy-CBD) [7]. THC binds to endocannabinoid receptors CB1 and CB2, acting as a partial agonist, and CBD affects the activity of various enzymes and receptors [7]. CBMs have been shown to have anti-spastic, anti-epileptogenic and anti-emetic activity, and there is strong evidence of their efficacy in neuropathic pain [8, 9]. Studies on CBM suggest clinical evidence in neuropsychiatric disorders, especially in reducing anxiety and psychotic symptoms, and in treating addiction to opioids and cocaine [10]. Still, more robust scientific evidence is needed to confirm those effects.

Despite a substantial increase in clinical trials (CT) on cannabinoid-based medications over the past decade (30 CT on CBM registered in 2013, 103 in 2023, ~250% increase), the total number of CT with CBM only represented ~0.02% of total CT registered around the world in 2023 [11, 12]. Of those, just a handful are focused on BPSD, with limited CT and little evidence on severely demented patients with comorbidities and advanced age [12].

Following encouraging results from a prior prospective observational study [13], we conducted the MedCanDem trial to evaluate the efficacy and safety of a THC/CBD oil in patients with severe dementia [14].

## Method

The MedCanDem study is a multicenter, randomised, double-blind, placebo-controlled AB/BA crossover trial of CBM in patients with behavioural and psychological troubles and pain in severe dementia disorder. The study was granted approval by the Geneva Ethics Committee (Commission Cantonale d’Éthique de la recherche approval number 2022-00999) and is registered on clinicaltrials.gov (NCT05432206) and the Swiss National Clinical Trials Portal (SNCTP 000005168). The study protocol was published [14]. This study was designed and reported in accordance with the CONSORT 2010 statement, extended version for reporting crossover clinical trials.

### Study population and settings

Participants were permanent residents aged 55 years and older in one of five long-term care facilities (LCTFs) in the Canton of Geneva, Switzerland, each representing a different study centre. The study took place from September 2023 to November 2024. Eligible participants were suffering from severe dementia of different origins, with a Clinical Dementia Rating (CDR) score of 3, a Neuropsychiatric Inventory score > 10 and consent provided by a representative in the medical field (art. 378 Swiss Civil Code). The underlying aetiology of the major neurocognitive disorder was not systematically classified, as major neurocognitive disorder does not necessarily imply Alzheimer’s disease and may also reflect vascular, mixed or other dementias. Patients were not included if they had any severe medical condition. All concomitant medications were allowed; other cannabinoids were excluded, and other psychotropic medications were excluded if their dosage necessitated regular adaptations.

### Study treatment

The study drug was a standardised cannabis oil—*Cannabis sativa* extractum oleatum normatum 10 mg tetrahydrocannabinol (THC) tot./ml—with 1% THC and 2% CBD. The active treatment, produced by Cannapharm AG (Switzerland), was odour and taste-masked with a 2% cinnamon essential oil. The placebo was a virgin hemp seed oil, odour masked with cinnamon bark oil, colour-adjusted with magnesium chlorophyll (E140). The investigational medicinal product was administered orally in drops, mixed with a spoonful of a fatty substance, such as yogurt or cake, or with a fruit compote.

The starting dosage was seven drops in the evening (2.5 mg THC/5 mg CBD), increased by seven drops at each step, up to a maximum of 56 drops (20 mg THC/40 mg CBD) a day, divided into two oral administrations (Table 1).

**Table 1:** The study titration schema, number of drops per day (and corresponding THC/CBD dosages for the active group)– From day 16 to 56, 56 drops/day.

DAY	Number of drops			mg THC/mg CBD
	Morning N	Evening N	Day total N	
1	0	7	7	2.5 mg THC/5 mg CBD
2	0	7	7	2.5 mg THC/5 mg CBD
3	7	7	14	5 mg THC/10 mg CBD
4	7	7	14	5 mg THC/10 mg CBD
5	7	14	21	7.5 mg THC/15 mg CBD
6	7	14	21	7.5 mg THC/15 mg CBD
7	14	14	28	10 mg THC/20 mg CBD
8	14	14	28	10 mg THC/20 mg CBD
9	14	21	35	12.5 mg THC/25 mg CBD
10	14	21	35	12.5 mg THC/25 mg CBD
11	21	21	42	15 mg THC/30 mg CBD
12	21	21	42	15 mg THC/30 mg CBD
13	21	28	49	17.5 mg THC/35 mg CBD
14	21	28	49	17.5 mg THC/35 mg CBD
15	28	28	56	20 mg THC/40 mg CBD

The standard titration was proposed, but dosages were monitored and adjusted reflecting the recommended approach to prescribing medical cannabinoids for other indications; all dosages between 2.5 mg THC/5 mg CBD and 20 mg THC/40 mg CBD per day were acceptable. Medication adherence was strictly monitored by the facility’s nurses, who recorded all administrations and any refusals on two different record systems. Also, the study pharmacist checked compliance with administration, comparing the two data sheets and bottle consumption. All usual concomitant medications and as-needed (PRN, *pro re nata*) medications were allowed; their prescription and management remained the responsibility of the treating physician.

The study was a crossover design with patients randomly assigned to the active treatment/placebo sequence or the reverse. The two periods were 8 weeks long, with a washout week planned in between them. The week washout was planned to combine data on CBD distribution half-life with the need to keep the study period as short as possible, given the complexity of the population and real-world settings. The treatment was abruptly stopped at the end of each period. Also, a final observation week was scheduled for the end of the study. Patients, relatives and study staff were blinded. The study treatment was provided in pre-filled kits containing two boxes: period 1 and period 2. Active treatment and placebo were placed in the boxes using a simple randomisation schema generated with a random number table, known only to the manufacturer and the statistician.

### Assessments

The primary endpoint was the delta-change in Cohen-Mansfield Agitation Inventory (CMAI) score relative to baseline [15]. This validated instrument, used to assess agitation in patients with dementia, comprises 29 items describing agitated behaviours, evaluated during a caregiver interview. Each item is rated on a 7-point Likert scale, ranging from 1 (never) to 7 (several times an hour), based on its frequency in the preceding week. The total CMAI score is calculated by summing all item scores, ranging from 29 to 203, yielding higher scores indicate greater agitation severity. The CMAI assesses multiple dimensions of agitation, including physically aggressive, physically non-aggressive and verbally agitated behaviours.

The secondary outcomes included changes from baseline on different scales, including the Neuropsychiatric Inventory (NPI) [16], which assesses other behavioural and psychological symptoms in patients with dementia. It evaluates 12 neuropsychiatric domains, rating them based on caregiver interview for frequency (1–4) and severity (1–3), with a domain score calculated as the product of frequency and severity (range 0–12). The total NPI score is obtained by summing all domain scores, yielding a possible range of 0–144, with higher scores indicating greater neuropsychiatric symptom burden. Another scale, the DOLOPLUS-2 pain score [17], is a validated observational instrument for assessing pain in older adults with severe cognitive impairment. It consists of 10 items grouped into three domains. The total DOLOPLUS-2 score is calculated by summing all item scores, yielding a possible range of 0–30, with higher scores indicating greater pain intensity. Also, changes in the most incapacitating daily activity (MIDA) and the most incapacitating behavioural trouble (MIBT), as defined by staff and/or relatives, were assessed on a 10-point visual scale, with higher values indicating a worse condition. The MIDA and MIBT were to be established at the first assessment, and the same activity/trouble was to be evaluated at all time points. Secondary endpoints assessment also included the Unified Parkinson’s Disease rating Item 22—rigidity scale (UPDRS), rated on a 0–4 scale, and the Team and Family Global Impression of Change (TGIC; FGIC) rating on a 7-point Likert-Type scale (1 very much improved to 7 very much worsened).

In addition, changes in the prescription and/or administration of psychotropic co-medications were systematically recorded and evaluated throughout the study. In particular, the use of PRN psychotropic medications was monitored and documented daily. These medications were routinely prescribed by physicians and administered by healthcare professionals only when a predefined and clinically justified need existed.

Safety assessments included the incidence and severity of adverse events (AEs) potentially related to study treatment, monitoring of weight, vital signs and blood formula, and daily blood pressure evaluation. The daily blood pressure assessment was implemented to monitor for potential changes that could lead to instability and additional falls. As per protocol, the mean arterial pressure (MAP; MAP = 1/3 systolic +2/3 diastolic) had to be determined before treatment started. If the daily MAP exceeded ±15% of the set MAP, this was considered an AE and investigated by physicians.

Secondary endpoints included exploratory evaluation of therapeutic drug monitoring (TDM) plasma concentrations of THC, CBD, 9-OH-THC and 9-COOH-THC, evaluation of metabolic phase-1 enzymatic activity (CYP1A2, CYP3A4, CYP2C19, CYP2D6, CYP2B6, CYP2C9), and possible variations in endocannabinoid plasma concentrations. This was conducted on blood samples collected at the beginning and end of both study periods, and analysis was conducted as per protocol.

Endpoint assessments were conducted by two trained study nurses and a physician. A panel of healthcare professionals who regularly cared for patients was interviewed as part of the scale assessments. A weekly exchange between nurses and the physician conducting the assessments was planned to align the management of the interviews. Assessments were completed on the first day of the study, after 4 weeks, and on the last day of each period. Also, a final evaluation was conducted after a week’s wash-out at the end of the study. Blood pressure was measured daily from the day of inclusion through the last day of the study, as well as all concomitant medications and changes in the prescription or administration of PRN medication. At the beginning and end of both study periods, blood samples were collected, patients were weighed and their temperatures were recorded.

### Data analysis

We planned to recruit 24 subjects based on the assumption of a treatment effect on the primary endpoint, a 20-point reduction from a baseline expected score of 70 with a between-patient standard deviation of 20 and a within-patient standard deviation of 15, and a power of 90% [14]. This assumption was based on a 2-year observational study in a similar population [13]. Randomisation to treatment sequences was 1:1, without stratification.

The complete analysis set was performed using Stata 18. The analysis followed an intention-to-treat (ITT) approach, including all randomised subjects as far as possible. No period effects or carry-over were assumed; in case of missing data, no subject replacement or imputation was performed.

According to the statistical analysis plan, changes from baseline in the CMAI score were compared intra-individually between the period under THC/CBD and the period under placebo, yielding the so-called cross-over difference. The matched-pairs *t-*test was used, with n–1 degrees of freedom. The change in CMAI is assumed to be approximately normally distributed, and no covariate adjustment was made. The same approach was used for secondary endpoints. TGIC scores at Day 56 were compared between active and placebo periods using within-subject cross-over differences. Independent *t-*tests were also performed on the primary and secondary endpoint results obtained during the first study period to assess between-treatment differences without the risk of carryover bias. PRN medication exposure was defined as the number of administrations per day, irrespective of pharmaceutical formulation. PRN medication use was analysed as total administration counts aggregated by treatment period and treatment sequence. Comparisons of PRN administration across treatment groups were performed using the chi-square test.

AEs/serious adverse events (SAEs) are presented as absolute numbers and percentages. Safety and tolerability were assessed (all randomly assigned participants who received any part of the trial treatment). Treatment-emergent adverse events were summarised by incidence, severity, causality and seriousness.

Blood sample analysis, TDM and endocannabinoid dosage results are presented descriptively, reflecting the raw data as collected and no inferential or statistical analyses were performed.

An independent data safety monitoring board monitored participant safety during the study; no results from intermediate analyses have been deemed necessary and performed.

### Data management

Study data were recorded and managed using the SecuTrial® electronic data capture system. Data quality was ensured through built-in validation checks and regular monitoring.

## Results

**Figure 1:**
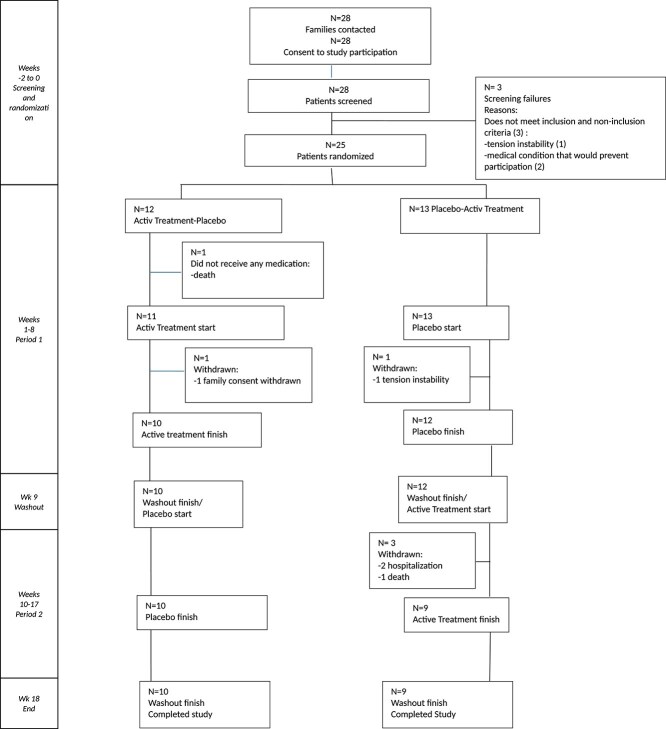
CONSORT flow diagram of the study.

The study was presented to the relatives or legal representatives of 28 preselected patients during group meetings, in which the study objectives and procedures were explained and questions addressed. Subsequently, study investigators met with interested families in individual consultations to discuss all aspects of the study in greater detail. All families or representatives of selected participants provided written informed consent (Figure 1).

Three participants were considered not eligible, and 25 residents of the 5 LCTF (68% female, 32% male) with an average age of 83 years were included. All participants had a *CDR score ≥ 3,* indicating severe cognitive impairment.

Participants were assigned to a consecutive kit numbered 1–25 in the order in which they were included in the study; 12 were assigned to the active-placebo group (A–P) and 13 to the placebo-active treatment group (P–A). Baseline characteristics differed between the two groups despite strict adherence to the protocol and randomisation procedures. Patients in the A–P group had higher CMAI baseline scores (Table 2).

**Table 2:** Baseline characteristics divided into A–P or P–A assignment groups. A = active treatment, P = placebo. The number of concomitant medications represents the number of prescribed administrations per day. In the last line, the number of patients who at the baseline presented with a prescription for PRN psychotropic medication. Between-group differences were assessed using independent-samples *t-*test for continuous variables and chi-square test for categorical variables.

Baseline characteristics				
		A–P	P–A	
Patients (n)		12	13	
				
		F/M	F/M	
Gender (n)		8/4	9/4	
				
		Mean (SD)	Mean (SD)	*P*-value
Age (years)		84.3 (4.3)	80.9 (6.3)	.13
				
Weight (Kg)		63.9 (13.6)	63.7 (10.4)	.97
				
CMAI score		84.9 (19.4)	65.1 (24.7)	**.04** ^a^
				
NPI score		82.1 (24.6)	64.5 (25.5)	.09
				
Doloplus score		9.7 (7.3)	6.5 (4.8)	.20
				
Concomitant medications (N/day)		7.8 (2.9)	9.1 (3.6)	.33
Concomitant psychotropic medications (N/day)		2.1 (1.4)	2.6 (1.4)	.38
			
		Yes/No N	Yes/No N	
Prescription of PRN psychotropic medications (number of patients)		10/2	7/6	.11

^a^Statistically significant (*P* < .05).

Patients were taking an average of 8 (A–P group = 7; P–A group = 9) concomitant medications, including all Anatomical Therapeutic Chemical (ATC) class of drugs (e.g. analgesics, laxatives, eye drops, cardiovascular medications, etc) and 2.5 psychotropic medications (A–P group = 2.1; P–A group = 2.6). Also, most patients (68%) had prescriptions for PRN psychotropic medications (A–P group 10 patients; P–A group 7 patients). Although prescribed as as-needed treatments, the psychotropic medications were, in fact, used regularly by all patients who had them prescribed.

One patient died after inclusion, was randomised, but didn’t receive the treatment, due to a sudden deterioration of his health. One family withdrew consent 28 days after inclusion and ended during the first period. They were both in the A–P group; the remaining 10 patients in this group completed the study as planned.

In the P–A group, one patient was excluded due to high arterial pressure instability a few days after starting the first period. Twelve patients ended the first period, and three were withdrawn during the second period; one frail and ill patient died for reasons unrelated to the study treatment, and two patients had to be hospitalised to treat infections ~6 weeks into the second period (SAE). Since the study team could not guarantee the same procedures and safety controls in hospital settings, these patients were excluded from the study. The nine remaining participants ended the study as planned.

### Daily dose

The proposed titration period was 15 days, and on average, it lasted 16 days for the active treatment and 15 days for the placebo. The dosage after titration was, on average, 51 ± 7 (SD) drops for the active treatment (17.85 mg THC/35.7 mg CBD ± 2.5 mg THC/5 mg CBD [SD]) and 54 ± 5 (SD) drops for the placebo.

A small number of patients occasionally refused treatment despite multiple administration attempts. Four patients missed 22 doses (0.46% of total planned doses; 12 in the active period and 10 in the placebo period); in all cases, treatment was resumed at the following scheduled administration.

### Efficacy

#### Primary endpoint—CMAI score

**Figure 2:**
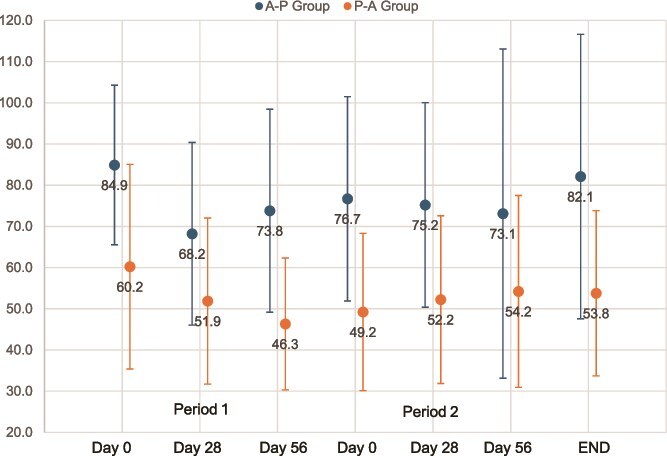
Variation of CMAI scores at Day 0, Day 28 and Day 56 during periods 1 and 2, and at the end of the study. CMAI scores are shown on the y-axis and are expressed as mean ± SD. A–P group: Active–placebo; P–A group: Placebo–active.

The primary endpoint assessed using the CMAI, did not show a statistically significant difference between the Active and Placebo periods. The mean cross-over difference was 5.0 (SD 33.8 – *P*-value of .53). In period 1, the scores on the CMAI decreased by a similar magnitude in both the active and placebo groups, with no statistically significant difference (*t-*test, *P* = .87) (Figure 2). A covariance analysis adjusted for baseline values was statistically significant (ANCOVA, *P* = .04) due to baseline imbalance between groups (effect of baseline score as covariate: *P* = .013), but the treatment effect was not statistically significantly different between active and placebo. In Period 2, the active group showed a slight increase in CMAI score whereas the placebo group decreased; the difference was not statistically significant (t-test, *P* = .38; ANCOVA, *P* = .54) (Table 3).

**Table 3:** Cross-over differences were calculated as within-subject differences between active and placebo periods. Paired *t-*tests were performed according to the statistical analysis plan. Inter-group comparisons by period were conducted using independent-samples t-tests as sensitivity analyses.

	n	Mean Cross-over Difference (A–P)	SD	Paired t-test *P*-value	Intergroup (period 1) *P*-value	Intergroup (period 2) *P*-value
**CMAI**	19	5.0	33.8	.53	.87	.38
**NPI**	19	1.8	49.6	.87	.82	.83
**Doloplus**	19	−2.4	9.4	.27	.29	.28
**TGIC**	19	0.21	2.47	.72	.40	.81

Inter-group analyses were performed using all available data per period (Period 1: n = 10/12; Period 2: n = 9/10).

*Abbreviations*: *CMAI, Cohen-Mansfield Agitation Inventory; NPI, Neuropsychiatric Inventory; TGIC, Team Global Impression of Change; A–P, Active-Placebo sequence; SD, standard deviation.*

#### Secondary endpoints—NPI, MIDA, MIBT, Doloplus pain score, UPDRS, TGIC, FGIC

The NPI mean cross-over difference was 1.8 (SD 49.6), with no statistically significant treatment effect (*P* = .87). Inter-group analyses showed no statistically significant changes between the active and placebo groups in either Period 1 (*P* = .82) or Period 2 (*P* = .83) (Table 3). The Doloplus mean cross-over difference was −2.4 (SD 9.4), with no statistically significant treatment effect (*P* = .27). The Doloplus pain score decreased in Period 1 in the active and placebo groups, with a larger magnitude in the active group; however, the between-group difference was not statistically significant (*t-*test, *P* = .29). In Period 2, the active group decreased the Doloplus pain score, whereas the placebo group increased it slightly; the difference was also not statistically significant (*t-*test, *P* = .28).

MIDA and MIBT were measured and recorded, but the data are unreliable because the care teams changed at each time point, and there was disagreement about the most incapacitating activity or behavioural trouble. UPDRS scores were recorded, but the data are limited to 9 patients because 15 patients did not present with rigidity at baseline or during the study at any time point. We can only draw limited conclusions due to the small sample size.

No statistically significant difference in TGIC scores was observed between active and placebo periods in the primary cross-over analysis (paired *t-*test *P* = .72). Inter-group comparisons by period did not show significant differences in Period 1 (*P* = .40) or Period 2 (*P* = .81), supporting the primary analysis. FGIC was not conducted because, as anticipated in the protocol, it was often difficult to contact families at the time of assessment, despite their collaboration and willingness to participate.

### Concomitant PRN (as-needed) psychotropic medications

The number of administered PRN psychotropic medications was significantly lower in the active treatment period (A–P group 43 PRN; P–A group 21 PRN) vs the placebo period (A–P group 99 PRN; P–A group 54 PRN). The overall rate of PRN medications was lower during the active treatment period (active = 0.06; placebo = 0.14 administrations per subject/day; chi-square test, *P* < .0001; Table 4).

**Table 4:** Total number of as-needed (PRN) medication administrations by treatment period and sequence. Period 1 and 2 = 56 days; washout 1 and 2 = 7 days.

Sequence	n	Active (n)	Placebo (n)	Washout after Active (n)	Washout after Placebo (n)
Active–Placebo	10	43	99	5	14
Placebo–Active	9	21	54	3	8

Few new drugs were prescribed during the study, only antibiotics. Some drugs were withdrawn, especially antihypertensive drugs, but overall prescriptions were stable throughout the study.

### Safety

All patients except one experienced MAP fluctuations, both above and below the established value. 107 (82.3%) of the 130 registered AEs were related to MAP. Of those, 71 events (66.3% of MAP events) were registered during the active treatment period; of those, 37 (52.1%) were tension below reference values, and 34 (47.9%) were above. Overall, average tension measurements (systolic, diastolic and MAP) were within reference values in both periods and did not change between the two periods in all patients (Table 5).

**Table 5:** Mean systolic blood pressure, diastolic blood pressure, mean arterial pressure (mmHg) and heart rate (bpm) by study period and by patient.

ID	Active				Placebo				Wash-out after Active				Wash-out after Placebo			
	SBP	DPB	MAP	HR	SBP	DPB	MAP	HR	SBP	DPB	MAP	HR	SBP	DPB	MAP	HR
1	132.4	79.4	97.1	78.2	128.9	76.8	94.2	78.0	138.4	82.4	101.1	84.4	129.7	77.4	94.9	75.9
2	122.8	67.1	85.6	70.4	124.6	71.5	89.2	75.1	124.3	68.4	87.0	80.1	117.2	66.8	83.6	68.8
3	129.0	73.6	92.1	63.7	131.9	78.1	96.0	71.9	140.3	81.0	100.8	65.9	137.1	74.9	95.6	67.9
4	127.9	78.5	94.9	87.1	133.0	82.9	99.6	91.3	129.5	78.3	95.3	88.6	133.6	84.4	100.8	87.7
5	143.3	70.8	94.9	83.0	140.5	68.9	92.8	82.0	138.4	71.6	93.9	79.9	137.7	75.6	96.3	80.1
6	114.9	67.9	83.6	63.4	120.4	69.4	86.4	64.2	120.4	69.4	86.4	64.2	116.4	71.3	86.3	64.3
7	122.3	75.6	91.2	82.9	119.2	77.2	91.2	89.5	119.2	77.2	91.2	89.5	115.0	72.3	86.5	101.0
8	128.0	78.6	95.0	76.9	127.9	80.9	96.6	81.2	128.9	75.7	93.4	74.4	126.3	79.1	94.9	79.7
9	120.7	68.6	86.0	66.5	127.2	74.3	91.9	71.1	135.0	78.9	97.6	66.3	117.7	68.3	84.7	65.7
10	115.2	66.0	82.4	75.6	119.4	71.3	87.4	78.5	113.0	67.8	82.9	66.8	119.6	71.7	87.7	74.7
11	122.4	70.3	87.7	80.2	123.9	69.6	87.7	85.1	117.1	67.6	84.1	83.7	131.1	73.3	92.6	79.6
12	125.2	73.2	90.5	91.5	132.1	80.3	97.6	91.2	132.7	77.2	95.7	83.5	128.7	77.0	94.2	91.0
13	140.9	74.8	96.8	65.5	130.2	75.3	93.6	68.7	151.3	79.9	103.6	65.0	153.0	78.0	103.0	65.3
14	128.6	69.2	89.0	75.3	131.5	71.4	91.4	75.2	144.3	75.4	98.4	77.4	133.9	71.9	92.5	80.0
15	114.5	60.9	78.7	69.7	116.6	62.3	80.4	69.0	115.8	61.2	79.4	64.0	111.4	62.6	78.9	73.1
16	118.7	69.7	86.0	69.2	118.1	70.2	86.2	72.5	126.3	82.3	97.0	67.6	124.4	76.0	92.1	74.0
17	119.6	69.3	86.1	70.8	125.2	72.7	90.2	73.4	128.4	77.1	94.2	75.7	123.6	70.9	88.4	69.1
18	123.4	66.1	85.2	67.8	115.1	64.3	81.2	67.6	130.0	67.6	88.4	73.0	122.1	66.0	84.7	65.9
19	127.9	80.7	96.4	73.7	127.5	74.5	92.2	77.0	133.0	84.9	100.9	74.3	121.7	72.2	88.7	76.7

Abbreviations: SBP, systolic blood pressure; DBP, diastolic blood pressure; MAP, mean arterial pressure; HR, heart rate.

A total of 23 AEs (excluding MAP values) and five SAEs were registered during the study (Table 6).

**Table 6:** Summary of AEs and SAEs by treatment period (active treatment, placebo and washout), coded using MedDRA terminology, including degree if severity.

					*THC/CBD oil*				*Placebo*				*Wash-out phases*			
*TOTAL AE*			*130*													
^a^ *SAE*			*5*					*3*				*1*				*1*
*Study participants N*			*25*													
		*%*	*N Events*	*N patients*	*Related*	*Severity*			*Related*	*Severity*			*Related*	*Severity*		
						*mild*	*mod.*	*sev.*		*mild*	*mod.*	*sev.*		*mild*	*mod.*	*sev.*
***System Organ Class*** ^b^	***Preferred Term*** ^b^															
																
*Blood and lymphatic system disorders*	*Anaemia unspecified*	*1.5*	*2*	*2*					*2*	*2*						
*Psychiatric disorders*	*Anxiety*	*0.7*	*1*	*1*	*1*		*1*									
	*Impulse control disorder*	*1.5*	*2*	*1*	*1*			***1*** ^a^	*1*			***1*** ^a^				
*Nervous system disorders*	*Confusional state*	*0.7*	*1*	*1*	*1*		*1*									
	*Psychomotor hyperactivity*	*2.3*	*3*	*1*	*1*		*1*		*1*		*1*		*1*		*1*	
	*Somnolence*	*3*	*5*	*5*	*3*		*3*		*2*		*2*					
*Skin and subcutaneous tissue disorders*	*Yellow nails syndrome*	*0.7*	*1*	*1*	*1*	*1*										
*Gastrointestinal disorders*	*Rectal fissure*	*0.7*	*1*	*1*	*0*			***1*** ^a^								
*General disorders and administration site conditions*	*Malaise*	*3.8*	*3*	*2*	*3*		*3*									
	*General physical health deterioration*	*1.5*	*2*	*2*	*0*			***1*** ^a^					*0*			***1*** ^a^
*Injury, poisoning and procedural complications*	*Fall*	*0.7*	*1*	*1*	*1*		*1*									
	*TOTAL MAP AEs*	*83.1*	*108*		*71*				*28*				*8*			
*Investigations*	*Mean arterial pressure increased*	*38.4*	*50*	*16*	*34*	*31*	*3*		*11*	*10*	*1*		*0*	*5*		
	*Mean arterial pressure decreased*	*43.8*	*58*	*17*	*37*	*8*	*29*		*17*	*13*	*4*		*2*	*4*		

^a^SAEs: serious adverse events.

^b^Adverse events were coded using the Medical Dictionary for Regulatory Activities (MedDRA), version 26.0.System Organ Class and Preferred Term (column headers) are shown in bold; individual terms are shown in italics.

The five SAEs were considered unrelated to study treatment but related to patients’ frailty and underlying comorbidities. One patient manifested twice an explosive intermittent behavioural disorder, once per period, and hospitalisation was necessary. In both cases, the disorder was attributed to an underlying urinary infection, causing great distress to the patient. Two participants died during the study: one person died before the beginning of the first period at the end of the inclusion phase due to a sudden and unexpected deterioration of the general health condition and wound superinfection. The other person died a few days into the second period—the active period—for a general decline in health conditions that had been noticed during the placebo period in the weeks preceding the event.

AEs were 15 (65.2%) in the active treatment phase, 6 (26.1%) in the placebo phase, and 2 (8.7%) in the washout period. They were mild to moderate events, all that could be expected with CBM use, and none led to exclusion from the study.

Heart rate was measured daily and remained stable throughout both study periods, with no increase observed at the start of the treatment phase during titration. A slight reduction in mean heart rate was observed during the treatment period compared with the placebo period, consistent with findings previously reported in younger patient populations (Table 5) [18].

Body weight and body temperature remained stable throughout the study and across both study periods (Appendix 1—Supplementary Figure 1). Haematological parameters were within the expected range for this population and remained stable throughout the study in both periods (Appendix 2—Supplementary Table 1).

### Exploratory endpoints

#### Therapeutic drug monitoring

The exploratory TDM analysis showed that the active compounds, THC and CBD, were absorbed and metabolised. After a 1-week washout period, metabolite residues were still detectable in patients’ plasma, consistent with the fact that in older adult populations the IMP half-life was longer than expected (Table 7) [19].

**Table 7:** Plasma concentrations of THC, 11-hydroxy-THC, THC-COOH, and CBD (ng/mL) measured at baseline and at Day 56 of each study period in individual patients.

Subject	Period	Day	THC (ng/mL)	THC-OH (ng/mL)	THC-COOH (ng/mL)	CBD (ng/mL)
1	P1	0	<0.5	<0.5	<0.5	<0.5
	P1	56	<0.5	3.08	61.1	2.88
	P2	0	< 0.5	0.93	0.90	2.22
	P2	56	< 0.5	0.68	< 0.5	0.75
2	P1	0	<0.5	<0.5	<0.5	<0.5
	P1	56	<0.5	<0.5	<0.5	<0.5
	P2	0	< 0.5	0.67	< 0.5	0.75
	P2	56	< 0.5	0.65	< 0.5	< 0.5
3	P1	0	<0.5	<0.5	<0.5	<0.5
	P1	56	1.91	12.8	71.1	6.73
	P2	0	1.08	2.15	5.31	3.56
	P2	56	< 0.5	0.79	< 0.5	0.74
4	P1	0	<0.5	<0.5	<0.5	<0.5
	P1	56	<0.5	<0.5	<0.5	<0.5
	P2	0	< 0.5	0.64	< 0.5	< 0.5
	P2	56	0.60	14	164	2.60
5	P1	0	<0.5	<0.5	<0.5	<0.5
	P1	56	<0.5	<0.5	<0.5	<0.5
	P2	0	< 0.5	0.63	< 0.5	< 0.5
	P2	56	0.47	7.68	186	4.46
6	P1	0	<0.5	<0.5	<0.5	<0.5
	P1	56	0.65	2.15	93	4.56
	P2	0	< 0.5	0.91	3.84	2.23
	P2	56	< 0.5	0.73	< 0.5	0.54
7	P1	0	<0.5	<0.5	<0.5	<0.5
	P1	56	0.65	4.91	302	7.35
	P2	0	< 0.5	1.08	104	4.89
	P2	56	< 0.5	0.80	< 0.5	0.66
8	P1	0	<0.5	<0.5	<0.5	<0.5
	P1	56	<0.5	<0.5	<0.5	<0.5
	P2	0	< 0.5	0.66	< 0.5	1.53
	P2	56	1.47	4.91	162	5.04
9	P1	0	<0.5	<0.5	<0.5	<0.5
	P1	56	0.62	7.04	131	3.74
	P2	0	< 0.5	1.50	31	2.48
	P2	56	< 0.5	0.80	< 0.5	0.60
10	P1	0	<0.5	<0.5	<0.5	<0.5
	P1	56	<0.5	<0.5	<0.5	<0.5
	P2	0	< 0.5	0.70	< 0.5	< 0.5
	P2	56	< 0.5	1.54	180	0.69
11	P1	0	<0.5	<0.5	<0.5	<0.5
	P1	56	<0.5	<0.5	<0.5	<0.5
	P2	0	< 0.5	0.69	< 0.5	< 0.5
	P2	56	< 0.5	1.83	195	4.33
12	P1	0	<0.5	<0.5	<0.5	<0.5
	P1	56	<0.5	<0.5	<0.5	<0.5
	P2	0	< 0.5	< 0.5	< 0.5	< 0.5
	P2	56	1.07	2.17	30	2.97
13	P1	0	<0.5	<0.5	<0.5	<0.5
	P1	56	<0.5	<0.5	<0.5	<0.5
	P2	0	< 0.5	< 0.5	< 0.5	< 0.5
	P2	56	2.05	7.22	151	5.22
14	P1	0	<0.5	<0.5	<0.5	<0.5
	P1	56	2.51	4.57	57.2	5.65
	P2	0	1.13	< 0.5	8.73	3.85
	P2	56	< 0.5	< 0.5	< 0.5	< 0.5
15	P1	0	<0.5	<0.5	<0.5	<0.5
	P1	56	<0.5	<0.5	<0.5	<0.5
	P2	0	< 0.5	< 0.5	< 0.5	< 0.5
	P2	56	< 0.5	3.04	119	3.43
16	P1	0	<0.5	<0.5	<0.5	<0.5
	P1	56	1.34	2.96	79.8	3.02
	P2	0	< 0.5	< 0.5	2.30	< 0.5
	P2	56	< 0.5	< 0.5	< 0.5	< 0.5
17	P1	0	<0.5	<0.5	<0.5	<0.5
	P1	56	2.32	4.53	134	5.35
	P2	0	1.83	< 0.5	21	4.75
	P2	56	< 0.5	< 0.5	< 0.5	< 0.5
18	P1	0	<0.5	<0.5	<0.5	<0.5
	P1	56	1.67	1.62	16.9	3.31
	P2	0	< 0.5	< 0.5	< 0.5	0.89
	P2	56	< 0.5	< 0.5	4.94	< 0.5
19	P1	0	<0.5	<0.5	<0.5	<0.5
	P1	56	<0.5	<0.5	<0.5	<0.5
	P2	0	< 0.5	< 0.5	< 0.5	< 0.5
	P2	56	< 0.5	< 0.5	99	1.71

Abbreviations: THC, Δ9-tetrahydrocannabinol; 11-OH-THC, 11-hydroxy-THC; THC-COOH, 11-nor-9-carboxy-THC; CBD, cannabidiol.

Values below 0.5 ng/mL indicate concentrations below the lower limit of quantification.

### Endocannabinoids dosages and phenotyping

Plasma concentrations of endocannabinoids (N-arachidonoylethanolamine [AEA], 2-arachidonoylglycerol [2-AG], N-palmitoylethanolamide [PEA] and N-oleoylethanolamide [OEA]) showed a modest increase in the active treatment group (6%–34%), remaining within physiological variability and not warranting further investigation (Appendix 2—Supplementary Table 2).

Phenotyping analysis using the Geneva cocktail probe drugs [20] resulted in expected range values without any phenotypic switches in excess after active treatment exposition. We didn’t register any sign of potential drug–drug interaction in our poly-medicated older adult population. The full report and analysis are the object of a dedicated publication in preparation (Appendix 2—Supplementary Table 3).

## Discussion

This is a unique crossover double-blind RCT on the use of CBM as add-on therapy for behaviour problems in multimorbid, polymedicated patients with severe dementia and stable medication, in a ‘real life’ setting of long-term older adults care.

The results show that both the placebo and treatment groups improved, with no significant difference in the primary endpoint. Still, we observed several interesting outcomes that suggest CBM might be an option for severe, treatment-resistant behavioural problems. The THC/CBD oil (1:2), slowly increased up to 20/40 mg, was well tolerated, showed no medication interactions, and no SAEs were related to the study drug. The treatment group significantly decreased PRN on-demand psychotropic medication.

Our study has several strong points. It was a randomised, double-blind, placebo-controlled trial conducted entirely in real-world settings, in long-term facilities outside the hospital. Also, the population was represented by multimorbid, polymedicated patients, and this is, to our knowledge, one of the few studies conducted on severely demented patients for whom prior deprescription of psychotropic medications could have been problematic. The MedCanDem study was well accepted by staff in the care settings, including the extensive training hours ahead of the study and the strict adherence to the study protocol. The facilities were outstanding collaborative and supportive of the study staff, providing all necessary assistance. The acceptance rate was extremely high (100%); none of the families declined to participate in the study, and only one withdrew consent. The dropout rate was acceptable in a fragile population and lower than the planned dropout rate in the protocol.

We have different hypotheses for why our initial hypothesis, based on a prior 2-year prospective observational study, could not be confirmed and why a potential positive effect might have been underestimated. The two groups differed significantly at baseline in the primary endpoint, with the A–P group presenting higher scores, despite the same clinical trial team overseeing all centres, the same selection criteria, and strict adherence to protocol and randomisation. Randomisation was not stratified on baseline severity, which may explain this imbalance despite correctly implemented allocation procedures and should be taken into consideration in further studies. Baseline characteristics indicated that patients presented a less severe clinical situation than in our observational study, especially regarding rigidity, almost absent in most patients included in the CT. The imbalance in the baseline severity between the two groups, combined with the substantial spontaneous improvements in most of the clinical scales in the two groups during the first period, may also have masked a possible effect of the active treatment. Consistently, the Period 1 ANCOVA signal reflected baseline severity rather than a treatment effect. Moreover, the observed cross-over difference of 5 points on the CMAI was well below the hypothesised 20-point threshold, and the 24% attrition rate further reduced statistical power.

Another limitation of the study’s interpretation is that the 1-week washout was too short, and some patients in the A–P group had plasma concentrations of active metabolites at the beginning of the placebo period, indicating a carry-over effect. The week washout was based on the usual THC and CBD half-life values in the adult population and on the need to keep the study timelines as short as possible. The slower metabolism in our older adult population was not anticipated [7], and no statistical analysis for carry-over effect was performed given the risk of low power of such tests [21]. Such carry-over analysis would tend to attenuate rather than inflate a true treatment effect, and Period 1 analysis, unaffected by carry-over, showed concordant results.

The primary endpoint and other assessment scales were linked to the subjectivity of a hetero evaluation. In the MedCanDem study, facilities staff were changing from one evaluation to the next, resulting in the same patient being evaluated from different perspectives at each time point. This might have affected the reliability of the results, even if an experienced, trained nurse conducted the scoring assessments. The same nurse conducted the prior prospective study, and she trained the other nurse and the physician who participated in assessments, which were often conducted jointly by two members of the study team.

This study could not demonstrate a significant difference between the active treatment period versus the placebo period, both groups improved. This ‘clinical study positive effect’ is well described in the literature, particularly in anxiety and depression clinical trials, and in another dementia-related agitation trial, possibly due to participants’ and investigators’ expectations and investment [22], as well as the regression-to-the-mean phenomenon. Also, across efficacy endpoints, substantial inter-individual variability was observed, as reflected by the large standard deviations relative to the mean cross-over differences, often several times larger than the estimated effect. This high variability indicates considerable heterogeneity in individual responses to treatment and suggests that potential treatment effects, if present, were inconsistent across subjects. A structural design constraint is that dementia aetiology was not systematically classified. Different pathologies—Alzheimer’s, vascular, Lewy body, frontotemporal or mixed, among others—may respond differently to cannabinoids, and future trials should consider stratification by dementia subtype. Still, in this institutionalised population, etiological diagnosis is frequently unavailable, difficult to investigate, and requiring it might restrict an already limited pool of eligible patients.

An important finding of this study is the significant reduction in the use of PRN psychotropic medications during the active treatment period. PRN psychotropic drugs, indicated for episodes of agitation, anxiety and insomnia, are part of crisis management. Their administration is based on the nurse’s assessment, including symptom severity, contextual factors and any non-pharmacological intervention already attempted, and requires rigorous traceability and medical oversight. The observed decrease in PRN psychotropic medication, whether reflecting a direct effect on agitation, a mild sedative effect of the active treatment, or a change in nursing staff’s perception and management of patient behaviour, results in fewer pharmacological interventions in the active period. Notably, PRN medications in this population typically include antipsychotics and benzodiazepines, which carry substantial risks in older adults with dementia; a reduction in their use therefore represents a potentially important clinical benefit in itself. PRN use may also represent a clinically meaningful indicator, possibly more sensitive than fixed-interval rating scales in capturing transient symptomatic fluctuations.

The nature of our multimorbid poly-medicated fragile population, the paucity of previous results, and the ‘real-world’ study settings did not allow us to stop already prescribed daily psychotropic medications. This also reflected the manifested physicians’ caution in changing ongoing therapies in a crossover and masked study. Moreover, some medications require deprescription gradual adjustments that could become problematic within the study. All those aspects, together with the routine PRN use in 68% of patients at baseline, possibly contributed to masking the efficacy of the cannabinoid-based treatment.

In comparison, in the phase 3 brexpiprazole clinical trial [23] for Alzheimer’s agitation, basic Alzheimer’s treatment was allowed, but all concomitant antipsychotics, mood stabilisers and anticonvulsant medications were stopped ahead of study start. It could be that the medication efficacy would have been less significant if psychotropic medications were allowed, as is the case in our study, which is closer to ‘real-life’ conditions and where a ‘ceiling effect’ on efficacy cannot be excluded [24].

This study provides a well-defined dosage and titration schema that ensures correct administration within established safety boundaries. The careful safety monitoring, particularly regarding blood pressure and AEs, was conducted daily throughout the 14 months, thanks to a reactive study team with study staff and investigators available to evaluate and respond promptly. This provides a large and valuable dataset in a population that remains scarcely studied and may serve as a reference for future clinical trials. Notably, the daily tension measurement, despite exhibiting high inter-individual variability and erratically falling outside the strict boundaries provided in the protocol, showed that mean tension and heart rate were stable during the study period, regardless of treatment.

An additional strength of this study is the reassuring finding that in this frail population, in whom chronic exposure to THC/CBD was systematically assessed, no phenotypic switch was observed, even if previous studies conducted in healthy volunteers after single or repeated doses suggested potential pharmacokinetic interactions [25, 26].

In the MedCanDem study, we proposed the study medication of THC/CBD oil as free treatment to patients willing to continue at the end of the trial. Treating physicians were to prescribe the treatment and ensure the follow-up, with the study team available to address any questions. Except for one, all representatives of participants who ended the study asked to continue the CBM. Six months after the patient’s last visit, all patients were continuing the THC/CBD oil, with 21% that could stop all other psychotropic medication.

## Conclusions

This study constitutes a significant step forward in the knowledge of cannabinoid-based medication for behavioural troubles in patients with severe dementia, despite the absence of demonstrated superiority over placebo. In a context where available pharmacological alternatives carry substantial safety concerns in this vulnerable population, demonstrating a favourable risk–benefit profile is itself a clinically relevant contribution. As one of the first clinical trials conducted in this population, it confirms the safety of this medication even in polymedicated patients and indirectly underscores the potential sparing effect on concomitant psychotropic drugs—a clinically valuable finding.

Several other lessons can be learned from our unique experience that can guide future research, including a clear titration schema to support prescribing physicians, the feasibility of conducting trials in LCTF—with careful planning to reduce the impact of staff shifts—and the relatively stable long-term blood pressure profile, suggesting that strict control may not be necessary.

Although further studies are needed to confirm efficacy, these data suggest a potential role for cannabinoid-based medication as a safe therapeutic alternative for behavioural and psychological symptoms of dementia, which can be initiated and maintained when a clinically meaningful response is achieved.

## Supplementary Material

Supplementary-material_afag239

## References

[1] Cao Q, Tan CC, Xu W et al. The prevalence of dementia: a systematic review and meta-analysis. J Alzheimer’s Dis 2020;73:1157–66. 10.3233/JAD-191092. 31884487

[2] Gianfredi V, Nucci D, Pennisi F et al. Aging, longevity, and healthy aging: the public health approach. Aging Clin Exp Res 2025;37:125. 10.1007/s40520-025-03021-8. 40244306 PMC12006278

[3] Radbruch L, De Lima L, Knaul F et al. Redefining palliative care-a new consensus-based definition. J Pain Symptom Manage 2020;60:754–64. 10.1016/j.jpainsymman.2020.04.027. 32387576 PMC8096724

[4] Day GS, Zayat R, Piura YD et al. Palliative care resource utilization in patients with prion disease. Alzheimer’s Dementia 2025;21:e102218. 10.1002/alz70858_102218. PMC12406364 40893060

[5] Watt JA, Porter J, Tavilsup P et al. Guideline recommendations on behavioral and psychological symptoms of dementia: a systematic review. J Am Med Dir Assoc 2024;25:837–846.e21. 10.1016/j.jamda.2024.03.007. 38640961

[6] Broers B, Bianchi F. Cannabinoids for behavioral symptoms in dementia: an overview. Pharmacopsychiatry 2024;57:160–8. 10.1055/a-2262-7837. 38447959

[7] Lucas CJ, Galettis P, Schneider J. The pharmacokinetics and the pharmacodynamics of cannabinoids. Brit J Clinical Pharma 2018;84:2477–82. 10.1111/bcp.13710. PMC6177698 30001569

[8] Quintero JM, Pulido G, Giraldo LF et al. A systematic review on cannabinoids for neuropathic pain administered by routes other than oral or inhalation. Plants 2022;11:1357. 10.3390/plants11101357. 35631782 PMC9145866

[9] Martinez-Paz C, García-Cabrera E, Vilches-Arenas Á. Effectiveness and safety of cannabinoids as an add-on therapy in the treatment of resistant spasticity in multiple sclerosis: a systematic review. Cannabis Cannabinoid Res 2023;8:580–8. 10.1089/can.2022.0254. 37057959

[10] Zhai X, Sarkar PR, Hill KP. Therapeutic use of cannabis and cannabinoids: benefits and risks. Polish Arch Internal Med published online 12 Sept 2025. 10.20452/pamw.17117. Epub 2025 Sep 12. PMID: 40938211. 40938211

[11] ClinicalTrials.gov [Internet]. Bethesda (MD): National Library of Medicine (US). Trends, Charts, Data. https://clinicaltrials.gov/about-site/trends-chartsClinicaltrials.gov (11 June 2025, date last accessed).

[12] Modaresi F, Talachian K. The characteristics of clinical trials on Cannabis and cannabinoids: a review of trials for therapeutic or drug development purposes. Pharm Med 2022;36:387–400. 10.1007/s40290-022-00447-7. 36357543

[13] Pautex S, Bianchi F, Daali Y et al. Cannabinoids for behavioral symptoms in severe dementia: safety and feasibility in a long-term pilot observational study in nineteen patients. Front Aging Neurosci 2022;14:957665. 10.3389/fnagi.2022.957665. 36247984 PMC9557769

[14] Bianchi F, Pautex S, Wampfler J et al. Medical cannabinoids for painful symptoms in patients with severe dementia: a randomized, double-blind cross-over placebo-controlled trial protocol. Front Pain Res 2023;4:1108832. 10.3389/fpain.2023.1108832. PMC10244760 37293434

[15] Cohen-Mansfield J, Deutsch LH. Agitation: subtypes and their mechanisms. Semin Clin Neuropsychiatry 1996;1:325–39. 10.1053/SCNP00100325. 10320435

[16] Cummings JL, Mega M, Gray K et al. The neuropsychiatric inventory: comprehensive assessment of psychopathology in dementia. Neurology 1994;44:2308–14. 10.1212/wnl.44.12.2308. 7991117

[17] Lefebvre-Chapiro S . The Doloplus-2 scale–evaluating pain in the elderly., pt 191-194. Eur J Palliat Care 2001;8:191-194.

[18] Jakob J, Stalder O, Kali T et al. The coronary artery risk development in young adults (CARDIA) study. Am J Med 2022;135:871–878.e14. 10.1016/j.amjmed.2022.01.057. 35245494

[19] Porter B, Marie BS, Milavetz G et al. Cannabidiol (CBD) use by older adults for acute and chronic pain. J Gerontol Nurs 2021;47:6–15. 10.3928/00989134-20210610-02. PMC8344100 34191653

[20] Bosilkovska M, Samer C, Déglon J et al. Evaluation of mutual drug–drug interaction within Geneva cocktail for cytochrome P450 phenotyping using innovative dried blood sampling method. Basic Clin Pharmacol Toxicol 2016;119:284–90. 10.1111/bcpt.12586. 27009433

[21] Senn SJ . Cross-over trials, carry-over effects and the art of self-delusion. Stat Med 1988;7:1099–101. 10.1002/sim.4780071010. 3206004

[22] Mora MS, Nestoriuc Y, Rief W. Lessons learned from placebo groups in antidepressant trials. Philos Trans R Soc B 2011;366:1879–88. 10.1098/rstb.2010.0394. PMC3130402 21576145

[23] Lee D, Slomkowski M, Hefting N et al. Brexpiprazole for the treatment of agitation in Alzheimer dementia: a randomized clinical trial. JAMA Neurol 2023;80:1307. 10.1001/jamaneurol.2023.3810. 37930669 PMC10628834

[24] Arslan J, Benke K. Statistical analysis of ceiling and floor effects in medical trials. Appl Biosci 2023;2:668–81. 10.3390/applbiosci2040042.

[25] Salcedo P, Volpe DA, Chaturbedi A et al. Clinical study to evaluate drug interactions of cannabidiol with citalopram and morphine in healthy adults. Clin Pharmacol Ther published online 1 Feb 2026. 10.1002/cpt.70219. Epub 2026 Feb 1. PMID: 41622700; PMCID: PMC12997498. PMC12997498 41622700

[26] Gorbenko AA, Post TE, Strugala PK et al. Low-dose cannabidiol increases plasma concentrations of amitriptyline: a clinical drug-drug interaction study. Br J Clin Pharmacol published online 15 Dec 2025. 10.1002/bcp.70415. Epub 2025 Dec 15. PMID: 41398552; PMCID: PMC13206195. PMC13206195 41398552

