# Supplementary material for: A randomised, double-blind, placebo-controlled crossover trial on cannabinoid-based medication for behavioural disorders in old patients with severe dementia: the study results
Source: Age Ageing. 2026 Aug 18;55(8):afag239. doi: 10.1093/ageing/afag239 (PMC13483985; doi:10.1093/ageing/afag239)
Supplement: Supplementary file 1 [file supplementary-material_afag239.zip › aa-26-0683-File002_afag239.docx]

**A randomized, double-blind, placebo-controlled crossover trial on cannabinoid-based medication for behavioral disorders in old patients with severe dementia: the study results.**

**Appendix 1**

**Supplementary Figure 1.** Variations in body weight, and body temperature during Period 1 (P1) and Period 2 (P2) in the Active–Placebo and Placebo–Active groups.
(A) Body weight. (B) Body temperature.

A1

A2

A1

(A) Body weight variations in the Active–Placebo (A1) and Placebo–Active (A2) groups during P1 and P2.

B2

B1

B1

(B) Body temperature variations in the Active–Placebo (B1) and Placebo–Active (B2) groups during P1 and P2.
