# Supplementary material for: A randomised, double-blind, placebo-controlled crossover trial on cannabinoid-based medication for behavioural disorders in old patients with severe dementia: the study results
Source: Age Ageing. 2026 Aug 18;55(8):afag239. doi: 10.1093/ageing/afag239 (PMC13483985; doi:10.1093/ageing/afag239)
Supplement: Supplementary file 1 [file supplementary-material_afag239.zip › aa-26-0683-File003_afag239.docx]

**A randomized, double-blind, placebo-controlled crossover trial on cannabinoid-based medication for behavioral disorders in old patients with severe dementia: the study results.**

**Appendix 2**

**Supplementary Tables 1,2,3.**

Supplementary Table 1. Individual hematological parameters

Supplementary Table 2. Individual plasma concentrations of endocannabinoids

Supplementary Table 3. Individual cytochrome P450 phenotyping results

**Supplementary Table 1. Individual hematological parameters**

| ID | Period | Day | Red Blood cell count | Hemoglobin | Hematocrit | Neutrophils | Neutrophils (absolute count) | Lymphocytes | Lymphocytes (absolute count) | Monocytes | Eosinophils | Basophils | Thrombocytes |
| --- | --- | --- | --- | --- | --- | --- | --- | --- | --- | --- | --- | --- | --- |
|  |  |  | 10^12^/L | g/L | % | % | G/l | % | G/l | % | % | % | G/l |
| 1 | P1 | 0 | 3.8 | 124 | 37.3 | 63.6 | 6.23 | 24.2 | 2.37 | 7.1 | 4.2 | 0.9 | 294 |
|  | P1 | 56 | 4.2 | 128 | 38.6 | 60.9 | 5.72 | 27.6 | 2.59 | 6.6 | 4.2 | 0.7 | 295 |
|  | P2 | 0 | 3.7 | 122 | 36.4 | 70.7 | 7 | 18.9 | 1.87 | 6.1 | 3.8 | 0.5 | 299 |
|  | P2 | 56 | 3.8 | 121 | 37.1 | 64.6 | 6.46 | 22.9 | 2.29 | 6.5 | 5 | 1 | 341 |
| 2 | P1 | 0 | 4.2 | 127 | 40.3 | 65.4 | 4.91 | 7.5 | 1.91 | 6.7 | 1.2 | 1.3 | 319 |
|  | P1 | 56 | 4.2 | 129 | 37.6 | 64.9 | 5.13 | 25.4 | 2.01 | 7 | 1.6 | 1.1 | 310 |
|  | P2 | 0 | 3.8 | 116 | 34.1 | 54.2 | 3.47 | 32.2 | 2.06 | 9.9 | 2.4 | 1.3 | 278 |
|  | P2 | 56 | 3.5 | 106 | 34 | 62 | 4.34 | 27 | 1.89 | 8 | 2 | 1 | 474 |
| 3 | P1 | 0 | 4.2 | 129 | 39.4 | 54 | 3.67 | 32.1 | 2.18 | 8.6 | 4.9 | 0.4 | 258 |
|  | P1 | 56 | 4.6 | 138 | 41.4 | 43.2 | 2.55 | 41.4 | 2.44 | 9.3 | 5.1 | 1 | 282 |
|  | P2 | 0 | 4.4 | 135 | 40.4 | 47.7 | 3.39 | 37.2 | 2.64 | 9.9 | 4.5 | 0.7 | 294 |
|  | P2 | 56 | 4.4 | 131 | 43 | 51 | 3.11 | 34 | 2.07 | 9 | 5 | 1 | 291 |
| 4 | P1 | 0 | 3.7 | 113 | 34.9 | 69.4 | 4.72 | 17.8 | 1.21 | 8.2 | 4.3 | 0.3 | 202 |
|  | P1 | 56 | 4.5 | 136 | 41.4 | 60.8 | 4.32 | 29.1 | 2.07 | 6.6 | 3.1 | 0.4 | 267 |
|  | P2 | 0 | 4.2 | 128 | 37.4 | 64.5 | 4.45 | 25.1 | 1.73 | 6.8 | 3.2 | 0.4 | 231 |
|  | P2 | 56 | 4.0 | 121 | 35.8 | 74 | 6.81 | 17.2 | 1.58 | 5.2 | 3.2 | 0.4 | 347 |
| 5 | P1 | 0 | 3.9 | 111 | 33.7 | 45.5 | 3.46 | 40.9 | 3.11 | 7.2 | 5.7 | 0.7 | 300 |
|  | P1 | 56 | 3.4 | 95 | 29.6 | 42 | 2.94 | 45.3 | 3.17 | 7.7 | 4.3 | 0.7 | 282 |
|  | P2 | 0 | 3.7 | 103 | 30.9 | 40.4 | 2.79 | 47.4 | 3.27 | 6.8 | 4.8 | 0.6 | 276 |
|  | P2 | 56 | 3.6 | 100 | 30 | 55.9 | 4.86 | 33.5 | 2.91 | 6.5 | 3.5 | 0.6 | 388 |
| 6 | P1 | 0 | 4.7 | 141 | 41.4 | 59.8 | 3.29 | 31 | 1.71 | 6.6 | 2.2 | 0.4 | 206 |
|  | P1 | 56 | 5.5 | 148 | 44.7 | 54.2 | 3.74 | 36.5 | 2.52 | 6.4 | 2.6 | 0.3 | 162 |
|  | P2 | 0 | 4.6 | 136 | 40.6 | 54.8 | 3.34 | 36.4 | 2.22 | 6.4 | 2.1 | 0.3 | 196 |
|  | P2 | 56 | 4.6 | 140 | 41.6 | 60.7 | 3.58 | 30.9 | 1.82 | 6.2 | 1.9 | 0.02 | 205 |
| 7 | P1 | 0 | 4.4 | 141 | 43.3 | 48.1 | 3.56 | 40.6 | 3 | 9.2 | 1.6 | 0.5 | 292 |
|  | P1 | 56 | 4.8 | 151 | 45.9 | 50 | 4.35 | 40.7 | 3.54 | 8.2 | 0.8 | 0.3 | 257 |
|  | P2 | 0 |  |  |  |  |  |  |  |  |  |  |  |
|  | P2 | 56 | 4.2 | 134 | 39.5 | 82 | 9.84 | 10.1 | 1.21 | 7.2 | 0.3 | 0.4 | 177 |
| 8 | P1 | 0 | 5.2 | 152 | 46.4 | 69.4 | 6.25 | 22.3 | 2.01 | 5.9 | 2.1 | 0.3 | 261 |
|  | P1 | 56 | 5.2 | 154 | 46 | 67.7 | 5.82 | 24.7 | 2.12 | 5.1 | 2 | 0.5 | 231 |
|  | P2 | 0 | 5.5 | 160 | 47.5 | 70.3 | 5.69 | 23.1 | 1.87 | 4.1 | 2 | 0.5 | 194 |
|  | P2 | 56 | 5.1 | 150 | 45.6 | 65 | 4.49 | 23.6 | 1.63 | 7.5 | 3.6 | 0.3 | 217 |
| 9 | P1 | 0 | 4.4 | 121 | 35.5 | 68.9 | 4.69 | 18.7 | 1.27 | 9.6 | 2.4 | 0.4 | 168 |
|  | P1 | 56 | 3.9 | 111 | 34 | 59.8 | 3.24 | 32.3 | 1.75 | 6.6 | 0.9 | 0.4 | 207 |
|  | P2 | 0 | 4.1 | 122 | 38 | 61.3 | 3.74 | 27.5 | 1.68 | 9.2 | 1.3 | 0.7 | 193 |
|  | P2 | 56 | 4.0 | 121 | 36.4 | 52.2 | 2.4 | 33.4 | 1.54 | 10.2 | 3.3 | 0.9 | 168 |
| 10 | P1 | 0 | 4.4 | 129 | 39.7 | 56.7 | 4.03 | 7.1 | 2.15 | 9.9 | 2.8 | 0.3 | 402 |
|  | P1 | 56 | 4.9 | 143 | 43.1 | 56.6 | 4.7 | 8.3 | 2.65 | 8.2 | 2.8 | 0.5 | 408 |
|  | P2 | 0 | 4.8 | 139 | 42.9 | 59.4 | 4.75 | 28.9 | 2.31 | 7.6 | 3.5 | 0.6 | 382 |
|  | P2 | 56 | 4.8 | 139 | 41.6 | 50.7 | 3.4 | 36.1 | 2.42 | 9.9 | 2.4 | 0.9 | 347 |
| 11 | P1 | 0 | 3.8 | 101 | 33.2 | 69.7 | 3.55 | 19.8 | 1.01 | 9.1 | 0.8 | 0.6 | 325 |
|  | P1 | 56 | 3.9 | 104 | 33.9 | 72.8 | 4.22 | 17.4 | 1.01 | 7.7 | 1.4 | 0.7 | 321 |
|  | P2 | 0 | 3.8 | 101 | 33.6 | 68.5 | 4.32 | 19.7 | 1.24 | 8.3 | 2.9 | 0.6 | 362 |
|  | P2 | 56 | 4.2 | 118 | 37.6 | 80.9 | 6.96 | 12.3 | 1.06 | 6.1 | 0.1 | 0.6 | 315 |
| 12 | P1 | 0 | 4.1 | 128 | 37.7 | 59.7 | 3.22 | 28.5 | 1.54 | 7 | 4.1 | 0.7 | 209 |
|  | P1 | 56 | 4.2 | 134 | 39.8 | 70 | 4.34 | 22 | 1.36 | 5.2 | 2.3 | 0.5 | 208 |
|  | P2 | 0 | 4.1 | 127 | 38.8 | 63.4 | 3.74 | 25.6 | 1.51 | 7.1 | 3.2 | 0.7 | 209 |
|  | P2 | 56 | 4.4 | 135 | 40.3 | 72.2 | 6.35 | 19.4 | 1.71 | 6.1 | 1.7 | 0.6 | 280 |
| 13 | P1 | 0 | 4.0 | 117 | 35.5 | 72.8 | 3.57 | 13.3 | 0.65 | 9.6 | 3.3 | 1 | 186 |
|  | P1 | 56 | 4.1 | 120 | 37.5 | 73.5 | 3.6 | 14.2 | 0.7 | 9.3 | 2.4 | 0.6 | 191 |
|  | P2 | 0 | 4.1 | 120 | 36.7 | 72.9 | 4.08 | 13.8 | 0.77 | 10.1 | 2.3 | 0.9 | 173 |
|  | P2 | 56 | 4.1 | 119 | 37.1 | 73 | 4.38 | 12.8 | 0.77 | 10.1 | 3.3 | 0.8 | 190 |
| 14 | P1 | 0 | 4.6 | 141 | 42.4 | 56.9 | 2.56 | 26.7 | 1.2 | 8.8 | 7.2 | 0.4 | 181 |
|  | P1 | 56 | 4.8 | 147 | 43.5 | 64.6 | 4.97 | 24.2 | 1.86 | 5.6 | 5.2 | 0.4 | 220 |
|  | P2 | 0 | 4.6 | 139 | 42 | 57.6 | 4.32 | 28.2 | 2.12 | 7.5 | 6.3 | 0.4 | 219 |
|  | P2 | 56 | 4.6 | 140 | 43.2 | 64.3 | 4.44 | 23.2 | 1.6 | 6.9 | 5 | 0.6 | 196 |
| 15 | P1 | 0 | 3.6 | 111 | 33 | 70.8 | 3.12 | 21.4 | 0.94 | 7.1 | 0 | 0.7 | 207 |
|  | P1 | 56 | 3.8 | 114 | 34.9 | 75.6 | 3.63 | 18 | 0.86 | 5.4 | 0.2 | 0.8 | 265 |
|  | P2 | 0 | 3.9 | 115 | 35.8 | 66.8 | 3.81 | 26.8 | 1.53 | 5.8 | 0.2 | 0.4 | 272 |
|  | P2 | 56 | 3.3 | 103 | 31.5 | 65.3 | 2.42 | 27.7 | 1.02 | 5.4 | 0.8 | 0.8 | 250 |
| 16 | P1 | 0 | 3.9 | 124.5 | 38.7 | 55.5 | 3.44 | 29 | 1.8 | 10 | 4.9 | 0.6 | 325 |
|  | P1 | 56 | 4.2 | 129 | 39.8 | 53.9 | 3.56 | 30.7 | 2.03 | 10.4 | 4.5 | 0.03 | 324 |
|  | P2 | 0 | 4.3 | 132 | 40.5 | 49.2 | 3.3 | 33.3 | 2.23 | 11.6 | 5.3 | 0.6 | 325 |
|  | P2 | 56 | 3.7 | 116 | 36.4 | 55.7 | 3.73 | 26.4 | 1.77 | 12.7 | 4.8 | 0.4 | 298 |
| 17 | P1 | 0 | 4.3 | 135 | 40.5 | 62.8 | 3.64 | 24 | 1.39 | 9.6 | 2.7 | 0.9 | 315 |
|  | P1 | 56 | 3.9 | 122 | 36 | 62.3 | 4.17 | 25.1 | 1.68 | 9.5 | 2.2 | 0.9 | 324 |
|  | P2 | 0 | 4.2 | 129 | 38.8 | 80.3 | 9.56 | 12.3 | 1.46 | 6.8 | 0.3 | 0.3 | 355 |
|  | P2 | 56 | 3.7 | 119 | 35.2 | 63.3 | 4.11 | 22 | 1.43 | 11.8 | 2 | 0.9 | 366 |
| 18 | P1 | 0 | 3.6 | 129 | 35.7 | 67.9 | 4.14 | 18.5 | 1.13 | 10.6 | 2.5 | 0.5 | 164 |
|  | P1 | 56 | 3.6 | 126 | 35.4 | 67.7 | 3.59 | 18.2 | 0.96 | 11.4 | 2.1 | 0.6 | 173 |
|  | P2 | 0 | 3.8 | 132 | 37.4 | 69.5 | 4.24 | 16.2 | 0.99 | 12.2 | 1.8 | 0.3 | 175 |
|  | P2 | 56 | 4.2 | 145 | 41 | 60.8 | 2.8 | 25 | 1.15 | 10 | 3.5 | 0.03 | 177 |
| 19 | P1 | 0 | 4.9 | 121 | 35.6 | 57.2 | 3.15 | 25.7 | 1.41 | 10 | 5.8 | 1.3 | 259 |
|  | P1 | 56 | 3.9 | 119 | 35.9 | 58.9 | 4.18 | 31.9 | 2.26 | 6.6 | 2 | 0.6 | 239 |
|  | P2 | 0 | 4.0 | 118 | 35.5 | 56.2 | 4.27 | 33.7 | 2.56 | 6.9 | 2.4 | 0.8 | 258 |
|  | P2 | 56 | 3.8 | 112 | 34 | 53.4 | 3.1 | 34.2 | 1.98 | 6.5 | 5 | 0.9 | 236 |

Individual hematological parameters for each participant at Day 0 and Day 56 of Period 1 (P1) and Period 2 (P2). Parameters include red blood cell count (10¹²/L), hemoglobin (g/L), hematocrit (%), and differential white blood cell counts expressed as relative percentages and absolute counts (G/L), where applicable.

**Supplementary Table 2. Individual plasma concentrations of endocannabinoids**

| ID | Period | Day | AEA | 2-AG | PEA | OEA |
| --- | --- | --- | --- | --- | --- | --- |
|  |  |  | (ng/mL) | (ng/mL) | (ng/mL) | (ng/mL) |
| 1 | P1 | 0 | 1.10 | 1.47 | 2.85 | 2.65 |
| 1 | P1 | 56 | 1.26 | 3.21 | 2.29 | 2.48 |
| 1 | P2 | 0 | 0.48 | 1.14 | 1.67 | 1.56 |
| 1 | P2 | 56 | 1.04 | 1.20 | 2.79 | 3.46 |
| 2 | P1 | 0 | 1.69 | 3.27 | 3.05 | 4.88 |
| 2 | P1 | 56 | 2.44 | 2.93 | 3.08 | 4.01 |
| 2 | P2 | 0 | 0.57 | 0.99 | 2.13 | 2.76 |
| 2 | P2 | 56 | 0.75 | 1.53 | 2.70 | 3.81 |
| 3 | P1 | 0 | 2.13 | 2.64 | 2.64 | 2.91 |
| 3 | P1 | 56 | 1.72 | 3.14 | 3.14 | 3.35 |
| 3 | P2 | 0 | 1.05 | 2.13 | 3.35 | 4.11 |
| 3 | P2 | 56 | 1.01 | 1.72 | 3.11 | 3.85 |
| 4 | P1 | 0 | 2.68 | 1.37 | 2.12 | 1.84 |
| 4 | P1 | 56 | 0.76 | 2.48 | 2.69 | 2.48 |
| 4 | P2 | 0 | 0.73 | 1.47 | 3.72 | 3.70 |
| 4 | P2 | 56 | 0.72 | 1.60 | 2.39 | 2.93 |
| 5 | P1 | 0 | 8.19 | 2.99 | 2.69 | 2.51 |
| 5 | P1 | 56 | 0.66 | 1.71 | 3.01 | 2.40 |
| 5 | P2 | 0 | 0.87 | 1.14 | 3.68 | 3.77 |
| 5 | P2 | 56 | 1.26 | 1.84 | 3.70 | 4.87 |
| 6 | P1 | 0 | 1.25 | 1.18 | 2.82 | 3.47 |
| 6 | P1 | 56 | 1.15 | 1.91 | 2.69 | 4.51 |
| 6 | P2 | 0 | 0.96 | 1.19 | 2.25 | 4.02 |
| 6 | P2 | 56 | 0.99 | 1.21 | 2.39 | 4.20 |
| 7 | P1 | 0 | 0.64 | 0.98 | 3.37 | 3.06 |
| 7 | P1 | 56 | 1.22 | 2.85 | 4.96 | 5.44 |
| 7 | P2 | 0 | 1.29 | 1.74 | 5.44 | 8.10 |
| 7 | P2 | 56 | 1.86 | 2.58 | 4.75 | 8.72 |
| 8 | P1 | 0 | 1.27 | 2.63 | 3.34 | 4.27 |
| 8 | P1 | 56 | 1.27 | 1.28 | 3.37 | 4.53 |
| 8 | P2 | 0 | 0.80 | 1.03 | 1.99 | 2.59 |
| 8 | P2 | 56 | 1.10 | 0.80 | 2.61 | 3.37 |
| 9 | P1 | 0 | 1.70 | 1.49 | 2.09 | 2.46 |
| 9 | P1 | 56 | 0.94 | 2.64 | 2.50 | 4.23 |
| 9 | P2 | 0 | 0.90 | 0.99 | 2.23 | 4.33 |
| 9 | P2 | 56 | 0.48 | 1.10 | 1.16 | 1.53 |
| 10 | P1 | 0 | 1.16 | 1.46 | 3.60 | 3.65 |
| 10 | P1 | 56 | 1.44 | 1.69 | 4.04 | 4.82 |
| 10 | P2 | 0 | 0.80 | 3.88 | 2.05 | 2.48 |
| 10 | P2 | 56 | 0.86 | 1.38 | 2.48 | 2.72 |
| 11 | P1 | 0 | 0.66 | 2.33 | 2.47 | 2.94 |
| 11 | P1 | 56 | 0.44 | 2.31 | 1.87 | 2.14 |
| 11 | P2 | 0 | 0.65 | 3.78 | 1.97 | 2.56 |
| 11 | P2 | 56 | 1.06 | 2.27 | 3.16 | 4.48 |
| 12 | P1 | 0 | 0.81 | 1.72 | 3.20 | 4.12 |
| 12 | P1 | 56 | 0.80 | 2.08 | 3.73 | 5.02 |
| 12 | P2 | 0 | 0.87 | 3.49 | 2.96 | 3.87 |
| 12 | P2 | 56 | 1.26 | 2.70 | 4.16 | 5.70 |
| 13 | P1 | 0 | 0.56 | 0.28 | 2.15 | 2.67 |
| 13 | P1 | 56 | 0.64 | 0.29 | 2.45 | 3.05 |
| 13 | P2 | 0 | 0.89 | 0.70 | 2.84 | 3.20 |
| 13 | P2 | 56 | 0.88 | 0.60 | 2.81 | 3.39 |
| 14 | P1 | 0 | 0.49 | 2.26 | 2.08 | 2.59 |
| 14 | P1 | 56 | 0.65 | 1.88 | 2.87 | 3.62 |
| 14 | P2 | 0 | 1.09 | 2.29 | 3.46 | 5.09 |
| 14 | P2 | 56 | 0.57 | 2.82 | 2.56 | 3.26 |
| 15 | P1 | 0 | 0.95 | 1.65 | 3.11 | 4.02 |
| 15 | P1 | 56 | 1.25 | 1.25 | 3.72 | 5.02 |
| 15 | P2 | 0 | 0.81 | 1.22 | 2.33 | 2.78 |
| 15 | P2 | 56 | 1.12 | 2.05 | 2.87 | 3.41 |
| 16 | P1 | 0 | 0.70 | 1.26 | 2.45 | 3.58 |
| 16 | P1 | 56 | 0.89 | 0.64 | 3.17 | 5.85 |
| 16 | P2 | 0 | 0.87 | 2.09 | 2.71 | 5.41 |
| 16 | P2 | 56 | 1.09 | 1.48 | 2.96 | 5.49 |
| 17 | P1 | 0 | 0.84 | 0.68 | 3.75 | 4.34 |
| 17 | P1 | 56 | 1.05 | 0.55 | 4.50 | 5.48 |
| 17 | P2 | 0 | 1.25 | 1.26 | 4.91 | 5.62 |
| 17 | P2 | 56 | 1.41 | 1.07 | 5.09 | 5.85 |
| 18 | P1 | 0 | 0.51 | 2.24 | 2.07 | 2.33 |
| 18 | P1 | 56 | 0.67 | 1.16 | 3.15 | 4.04 |
| 18 | P2 | 0 | 0.89 | 1.43 | 2.56 | 3.55 |
| 18 | P2 | 56 | 0.92 | 2.13 | 2.37 | 2.98 |
| 19 | P1 | 0 | 0.36 | 0.64 | 1.58 | 2.22 |
| 19 | P1 | 56 | 0.75 | 0.84 | 2.63 | 3.72 |
| 19 | P2 | 0 | 0.90 | 1.66 | 2.43 | 3.37 |
| 19 | P2 | 56 | 0.93 | 1.60 | 2.75 | 3.43 |

Individual plasma concentrations of endocannabinoids at Day 0 and Day 56 of Period 1 (P1) and Period 2 (P2). Anandamide (AEA), 2-arachidonoylglycerol (2-AG), palmitoylethanolamide (PEA), and oleoylethanolamide (OEA) concentrations are reported in ng/mL.

**Supplementary Table 3. Individual cytochrome P450 phenotyping results**

| ID |  |  | CYP1A2 | CYP2C19 | CYP2D6 | CYP3A4 | CYP2B6 | CYP2C9 |
| --- | --- | --- | --- | --- | --- | --- | --- | --- |
|  | Period | Day | Para/CAF | OH-OPZ/OPZ | DOR/DEM | OH-MDZ/MDZ | OH-BUP/BUP | OH-FLU/FLU |
| 1 | P1 | 0 | 0.17 | ND | 0.41 | 0.58 | 1.99 | 0.04 |
| 1 | P1 | 56 | 0.21 | 0.50 | 0.81 | 0.79 | 3.04 | 0.06 |
| 1 | P2 | 0 | 0.22 | 0.92 | 0.77 | 0.70 | 2.71 | 0.05 |
| 1 | P2 | 56 | 0.20 | 0.61 | 0.53 | 0.71 | 2.16 | 0.07 |
| 2 | P1 | 0 | 0.32 | ND | 0.65 | 0.54 | 5.15 | 0.02 |
| 2 | P1 | 56 | 0.42 | 0.77 | 0.72 | 0.94 | 5.07 | 0.01 |
| 2 | P2 | 0 | 0.23 | 0.72 | 0.83 | 0.84 | 2.64 | 0.03 |
| 2 | P2 | 56 | 0.28 | 0.60 | 0.91 | 0.67 | 2.64 | 0.03 |
| 3 | P1 | 0 | 0.15 | ND | 0.06 | 0.64 | 3.10 | 0.01 |
| 3 | P1 | 56 | 0.10 | 0.48 | 0.06 | 0.57 | 3.98 | 0.01 |
| 3 | P2 | 0 | 0.12 | 0.56 | 0.01 | 0.90 | 3.22 | 0.02 |
| 3 | P2 | 56 | 0.10 | 0.34 | 0.04 | 0.78 | 3.26 | 0.01 |
| 4 | P1 | 0 | 0.30 | ND | 2.21 | 0.94 | 1.06 | 0.02 |
| 4 | P1 | 56 | 0.26 | ND | 1.94 | 1.21 | 1.27 | 0.05 |
| 4 | P2 | 0 | 0.28 | 0.23 | 1.38 | 1.08 | 1.13 | 0.04 |
| 4 | P2 | 56 | 0.27 | 0.08 | 2.84 | 1.57 | 0.67 | 0.03 |
| 5 | P1 | 0 | 0.19 | 0.24 | 0.26 | 0.77 | 4.43 | 0.10 |
| 5 | P1 | 56 | 0.17 | 0.47 | 0.25 | 0.57 | 4.12 | 0.16 |
| 5 | P2 | 0 | 0.21 | 0.27 | 0.29 | 0.88 | 4.64 | 0.16 |
| 5 | P2 | 56 | 0.40 | ND | 0.40 | 0.95 | 0.92 | 0.17 |
| 6 | P1 | 0 | 0.37 | 0.49 | 0.48 | 0.11 | 0.21 | ND |
| 6 | P1 | 56 | 0.27 | 0.62 | 0.14 | 0.22 | 0.21 | ND |
| 6 | P2 | 0 | 0.21 | 0.30 | 0.01 | 0.97 | 1.43 | 0.08 |
| 6 | P2 | 56 | 0.24 | 0.20 | 0.44 | 0.26 | 5.07 | ND |
| 7 | P1 | 0 | 0.53 | 1.41 | 4.49 | 1.59 | 5.80 | 0.15 |
| 7 | P1 | 56 | 0.35 | 0.42 | 7.62 | 2.15 | 7.47 | 0.12 |
| 7 | P2 | 0 | 0.26 | 0.31 | 12.39 | 3.99 | 10.59 | 0.12 |
| 7 | P2 | 56 | 0.35 | 0.46 | 3.16 | 1.66 | 6.27 | 0.16 |
| 8 | P1 | 0 | 0.44 | 0.50 | 1.36 | 0.73 | 5.78 | 0.01 |
| 8 | P1 | 56 | 0.47 | 0.77 | 1.69 | 0.62 | 4.02 | 0.05 |
| 8 | P2 | 0 | 0.39 | 1.03 | 4.53 | 1.02 | 2.84 | 0.03 |
| 8 | P2 | 56 | 0.41 | 0.32 | 1.76 | 0.94 | 2.39 | 0.05 |
| 9 | P1 | 0 | 0.32 | 0.40 | 1.24 | 0.84 | 2.93 | 0.05 |
| 9 | P1 | 56 | 0.30 | 0.25 | 0.77 | 0.72 | 0.97 | 0.05 |
| 9 | P2 | 0 | 0.33 | ND | 0.34 | 0.69 | 1.15 | 0.03 |
| 9 | P2 | 56 | 0.25 | 0.28 | 0.85 | 0.84 | 1.49 | 0.06 |
| 10 | P1 | 0 | 0.48 | 0.97 | 1.07 | 0.78 | 5.64 | 0.03 |
| 10 | P1 | 56 | 0.31 | 1.46 | 1.64 | 1.46 | 4.11 | 0.05 |
| 10 | P2 | 0 | 0.32 | 0.76 | 1.66 | 1.23 | 1.34 | 0.01 |
| 10 | P2 | 56 | 0.32 | 0.86 | 1.43 | 2.21 | 5.18 | 0.05 |
| 11 | P1 | 0 | 0.21 | 0.91 | 0.80 | 0.56 | 10.91 | 0.10 |
| 11 | P1 | 56 | 0.45 | 2.70 | 0.80 | 0.11 | 17.81 | 0.13 |
| 11 | P2 | 0 | 0.23 | 1.05 | 1.48 | 0.77 | 1.10 | 0.11 |
| 11 | P2 | 56 | 0.24 | 0.51 | 1.20 | 0.63 | 5.34 | 0.14 |
| 12 | P1 | 0 | 0.30 | ND | 0.41 | 0.74 | 15.88 | 0.03 |
| 12 | P1 | 56 | 0.28 | 1.67 | 0.52 | 1.03 | 15.77 | 0.03 |
| 12 | P2 | 0 | 0.35 | ND | 0.57 | 1.07 | 10.61 | 0.04 |
| 12 | P2 | 56 | 0.21 | ND | 0.45 | 0.48 | 3.56 | 0.03 |
| 13 | P1 | 0 | 0.14 | 0.37 | 0.12 | 0.20 | 0.06 | 0.02 |
| 13 | P1 | 56 | 0.18 | 0.26 | 0.76 | 1.12 | 2.59 | 0.04 |
| 13 | P2 | 0 | 0.18 | 0.14 | 0.92 | 1.40 | 3.52 | 0.05 |
| 13 | P2 | 56 | 0.19 | 0.07 | 0.71 | 1.46 | 2.56 | 0.06 |
| 14 | P1 | 0 | 0.38 | 0.50 | 0.67 | 0.32 | 8.89 | 0.06 |
| 14 | P1 | 56 | 0.15 | 0.07 | 0.57 | 0.29 | 2.98 | 0.02 |
| 14 | P2 | 0 | 0.16 | ND | 1.71 | 0.33 | 7.24 | 0.08 |
| 14 | P2 | 56 | 0.18 | 0.08 | 1.84 | 0.62 | 5.26 | 0.07 |
| 15 | P1 | 0 | 0.14 | 0.27 | 0.66 | 0.87 | 4.47 | 0.05 |
| 15 | P1 | 56 | 0.16 | 0.25 | 0.84 | 1.15 | 4.29 | 0.06 |
| 15 | P2 | 0 | 0.19 | 0.07 | 0.54 | 1.09 | 4.31 | 0.08 |
| 15 | P2 | 56 | 0.19 | 0.17 | 0.77 | 1.60 | 4.52 | 0.09 |
| 16 | P1 | 0 | 0.22 | 0.96 | 1.08 | 1.14 | 4.01 | 0.10 |
| 16 | P1 | 56 | 0.29 | 1.17 | 0.65 | 0.77 | 4.11 | 0.11 |
| 16 | P2 | 0 | 0.32 | 2.05 | 0.57 | 1.13 | 2.33 | 0.10 |
| 16 | P2 | 56 | 0.37 | 0.72 | 1.20 | 1.54 | 0.96 | 0.12 |
| 17 | P1 | 0 | 0.23 | 0.94 | 1.19 | 1.40 | 4.23 | 0.06 |
| 17 | P1 | 56 | 0.46 | 0.39 | 0.97 | 1.76 | 6.52 | 0.06 |
| 17 | P2 | 0 | 0.25 | 0.60 | 1.17 | 1.97 | 6.67 | 0.06 |
| 17 | P2 | 56 | 0.46 | ND | 0.62 | 2.36 | 2.73 | 0.08 |
| 18 | P1 | 0 | 0.21 | 0.69 | 1.33 | 1.35 | 6.43 | 0.04 |
| 18 | P1 | 56 | 0.20 | 0.72 | 1.78 | 1.21 | 6.10 | 0.06 |
| 18 | P2 | 0 | 0.23 | ND | 1.90 | 3.14 | 2.80 | 0.07 |
| 18 | P2 | 56 | 0.31 | 1.23 | 1.51 | 0.98 | 2.71 | 0.09 |
| 19 | P1 | 0 | 0.16 | 0.36 | 0.04 | 0.71 | 2.79 | 0.06 |
| 19 | P1 | 56 | 0.10 | 0.15 | 0.03 | 0.49 | 3.81 | 0.02 |
| 19 | P2 | 0 | 0.08 | 0.16 | 0.02 | 1.30 | 1.19 | 0.05 |
| 19 | P2 | 56 | 0.26 | 0.16 | 0.12 | 1.01 | 2.28 | 0.07 |

Individual cytochrome P450 phenotyping results at Day 0 and Day 56 of Period 1 (P1) and Period 2 (P2), expressed as metabolic ratios for CYP1A2 (paraxanthine/caffeine), CYP2C19 (5-hydroxyomeprazole/omeprazole), CYP2D6 (dextrorphan/dextromethorphan), CYP3A4 (1-hydroxymidazolam/midazolam), CYP2B6 (hydroxybupropion/bupropion), and CYP2C9 (4-hydroxyflurbiprofen/flurbiprofen). ND: not detected.
